# Ring-like N-fold Models of Aβ_42_ fibrils

**DOI:** 10.1038/s41598-017-06846-0

**Published:** 2017-07-26

**Authors:** Wenhui Xi, Ulrich H. E. Hansmann

**Affiliations:** Department of Chemistry and Biochemistry, University of Oklahoma, Norman, Oklahoma 73019 USA

## Abstract

When assembling as fibrils Aβ_40_ peptides can only assume U-shaped conformations while Aβ_42_ can also arrange as S-shaped three-stranded chains. We show that this allows Aβ_42_ peptides to assemble pore-like structures that may explain their higher toxicity. For this purpose, we develop a scalable model of ring-like assemblies of S-shaped Aβ_1–42_ chains and study the stability and structural properties of these assemblies through atomistic molecular dynamics simulations. We find that the proposed arrangements are in size and symmetry compatible with experimentally observed Aβ assemblies. We further show that the interior pore in our models allows for water leakage as a possible mechanism of cell toxicity of Aβ_42_ amyloids.

## Introduction

**Figure 1:**
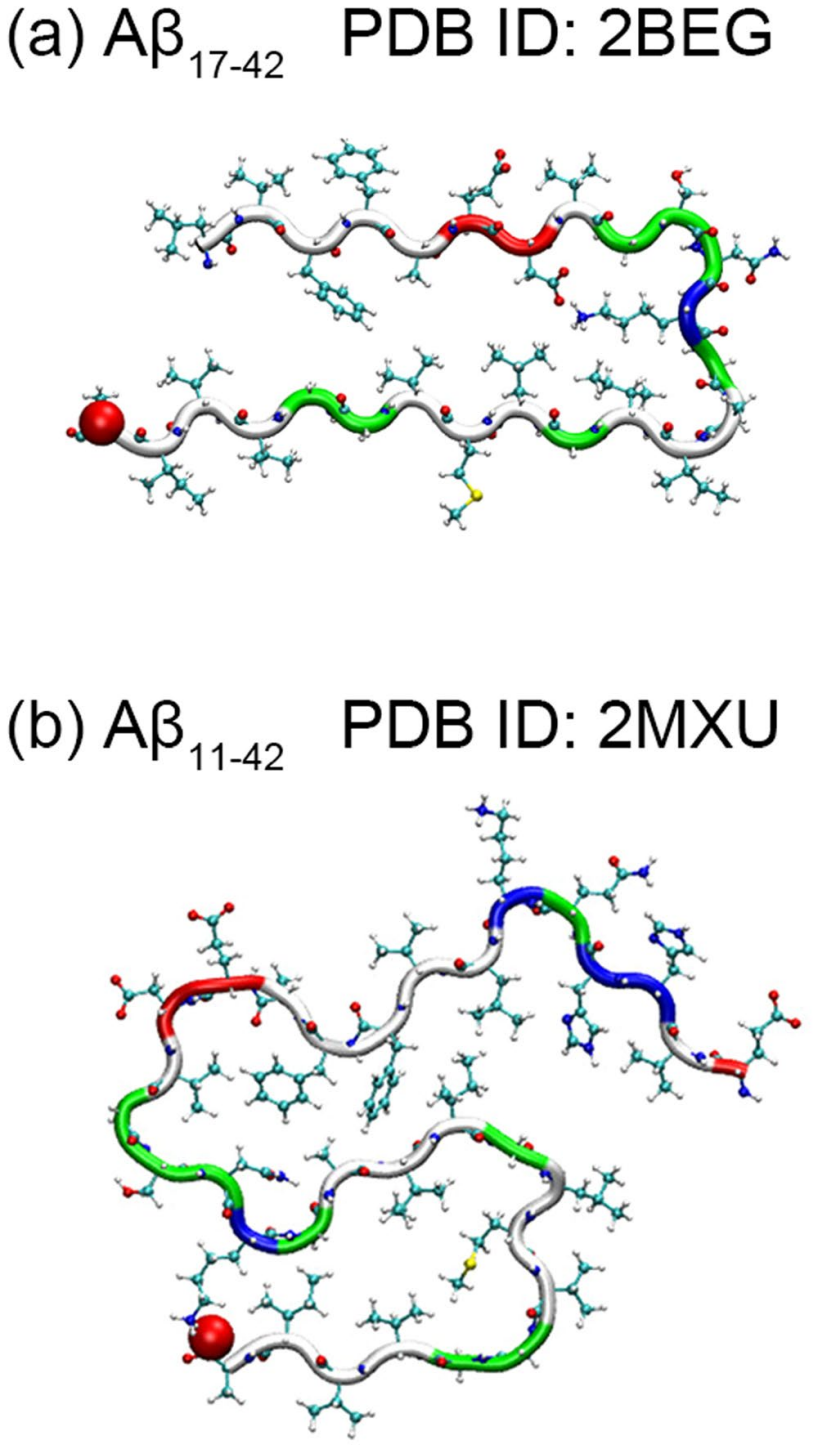
Polymorphism in Aβ fibrils. Single fold U-shaped (**a**) and S-shaped (**b**) Aβ_42_ fibril chains.

**Figure 2:**
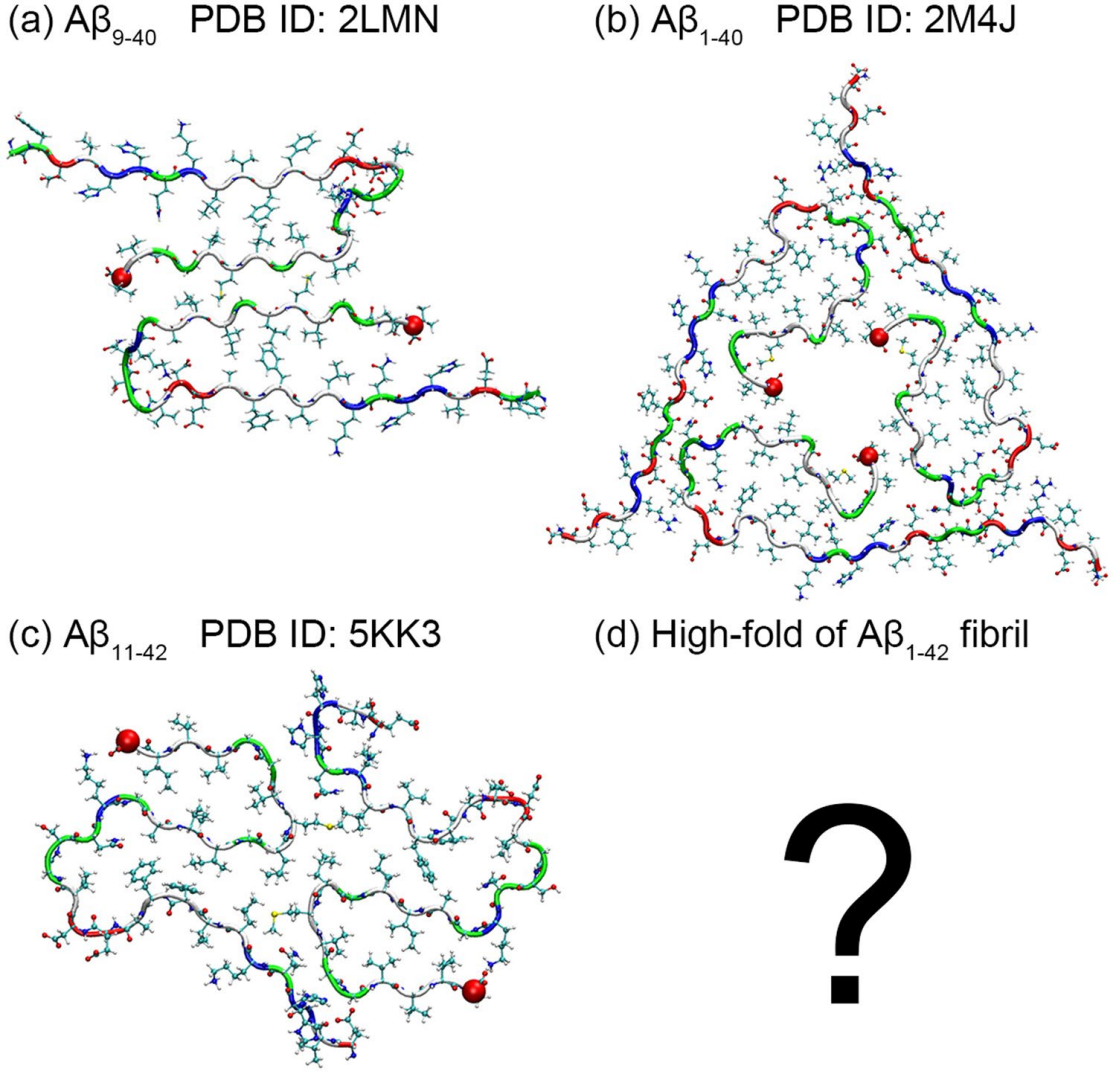
Polymorphism in Aβ fibrils. Top row: Two-fold (**a**) and Three-fold (**b**) U-shaped Aβ_40_ fibril chains; bottom row: two-fold S-shaped Aβ_42_ fibril chains (**c**); higher-fold fibril arrangements have not yet been observed experimentally.

The brains of patients with Alzheimer’s disease are characterized by the presence of amyloid fibrils that are made out of amyloid-β (Aβ) peptides, most commonly Aβ_1–40_ peptides but also the more toxic Aβ_1–42_ species^1–3^. Prerequisite for the emergence of mature fibrils is the self-assembly of monomers into a seed nucleus^4^. In this process, smaller oligomers are formed that appear to be the neurotoxic agents leading to memory loss in the case of Alzheimer’s disease^5, 6^. Monomers, oligomers and amyloid fibrils exist in an equilibrium of interchanging structures characterized by polymorphism^7–11^. As differences in molecular structure correlate with cell toxicity and speed of disease progress, it is important to characterize the structure of amyloids^11, 12^. At least five different structures of Aβ_1–40_ amyloid fibrils have been resolved by solid state NMR (ss-NMR), all sharing as a common motif that the individual chains form two β-strands connected by a loop region^7–11^. The situation is more complex for fibrils made out of Aβ_1–42_ peptides. Earlier low-resolution models exhibited the same U-shape motif, shown in Fig. 1a, where two β-strands made of residues 18–26 and residues 31–42 are connected by a loop, and the arrangement is stabilized by salt bridges between residues D23–K28^8^. However, a number of recent studies demonstrated the possibility of S-shaped chain arrangements^13–17^, see Fig. 1b, with the N-terminal strand β1 made of residues 12–18, the central strand β2 of residues 24–33, and the C-terminal strand β3 of residues 36–40^13^. Note that here, as in Fig. 2a and c, some N-terminal residues are not shown as the N-terminus is flexible and the structure could not be resolved for all its residues from the ss-NMR experiments. We have recently shown that this S-shaped structure is not stable in assemblies of Aβ_1–40_ peptides^18^. This is not because the lack of the β2 and β3 strand connecting salt bridge between the side chain of residue K28 and the main chain atoms of residue A42 that cannot be formed in Aβ_1–40_ peptides, but because of the lack of hydrophobic contacts that the C-terminal residues I41 and A42 form in Aβ_1–42_ peptides^18^.

It is tempting to assume that the higher toxicity of Aβ_1–42_ peptides is related to their ability to form this S-shaped motif. Such a correlation could arise if S-shaped Aβ_1–42_ peptides can assemble into structures that are either not possible, or less stable, than the assemblies formed by U-shaped Aβ chains. On first look this does not seem to be the case as the recently resolved S-shaped fibril structures^15, 16^ (Fig. 2c) have a similar two-fold packing as seen in the earlier determined Aβ_1–42_-fibrils with U-shaped chains and in most of the Aβ_1–40_ fibril structures deposited in the Protein Data Bank, see, for instance Fig. 2a. However, the only *patient-derived* structure in the Protein Data Bank has the chains arranged with a three-fold symmetry^11^ (Fig. 2b). Experimentally observed pore-like Aβ assemblies in membranes^19, 20^ also suggest the importance of ring-like arrangements for the disease mechanism. For instance, oligomers of Aβ_1–42_ with four to six-fold symmetry that can insert into membranes have been found by atomic force microscopy^19^, and similarly, β-barrel-like dodecamers that have pores have been proposed to act as ion channel in membranes^20^. Another example are the non-pore forming cylindrin-like out-of-register oligomers of Aβ^21, 22^ that are also toxic and related to membrane disruption. These and other oligomer models of Aβ that have been summarized in a recent review^23^ suggest looking for Aβ assemblies with three-fold and higher-fold symmetries as potential disease-causing agents. U-shaped Aβ chains can, because of steric constraints, only assemble into structures with N-fold symmetry of N ≤ 3, but the steric constraint argument does not hold for S-shaped chains. Hence, Aβ_1–42_ peptides, being unlike Aβ_1–40_ peptides able to assume a S-shaped form, may be able to form such pore-like higher-fold assemblies, causing them to be more cytotoxic.

The experimentally determined fibrils of S-shaped Aβ_1–42_ peptides have a 2-fold symmetry where the chains are packed through inter-chain contacts between residues M35 and either L17 or Q15, see Fig. 2c ^15^. Such packing of chains cannot be extended to assemblies with three and higher-fold symmetry. However, we have shown in previous work^18^ that S-shaped Aβ_1–42_ peptides can also pack by forming inter-chain salt-bridges between residue K16 and residues E22 or D23, an arrangement that is scalable to higher-fold packing. Hence, in the present paper, we propose a scalable model of ring-like assemblies of S-shaped Aβ_1–42_ chains based on the packing proposed by us in our previous work^18^. Using molecular dynamics simulations, we investigate the stability and properties of these assemblies as function of size. For the case of three-fold fibril arrangements, we compare these models with the patient-derived Aβ_1–40_ fibrils which also have three-fold symmetry (Fig. 2b). We find that the proposed arrangements are in size and symmetry compatible with experimentally observed fatty-acid-catalyzed oligomers and with pore-forming Aβ assemblies seen in membranes^19, 24^. We conjecture that the ability to form these arrangements with high N-fold symmetry may explain the high toxicity of Aβ_1–42_ amyloids.

## Results

### Comparison of our two-fold assemblies with experimentally resolved models of Aβ_42_

**Figure 3:**
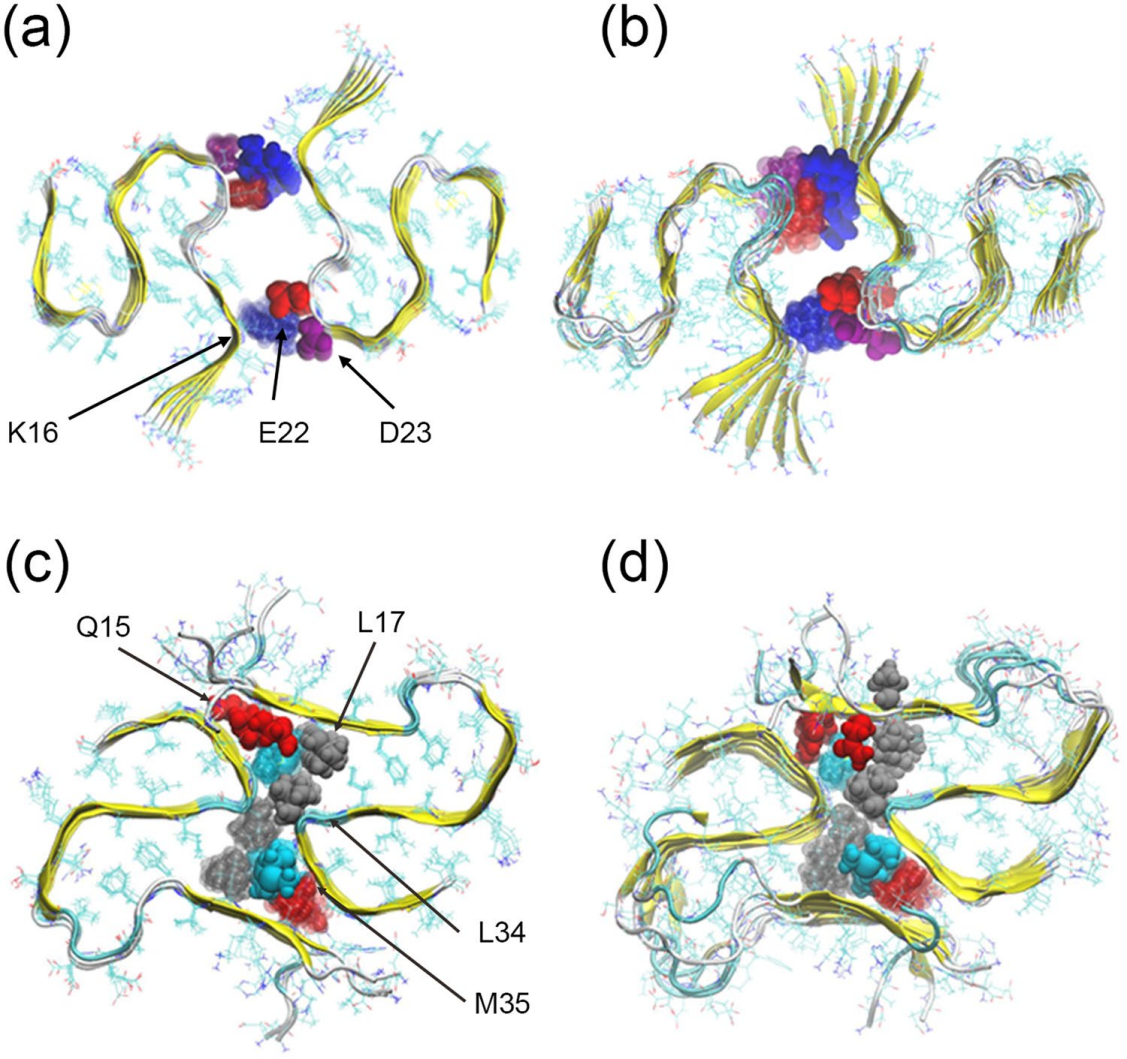
Conformation of our two-fold model and the JACS model. Initial (**a**) and final (**b**) conformation (after 200 ns of molecular dynamics simulation) of the two-fold model of Aβ_11–42_ predicted by us in previous work^18^. The corresponding configurations for the two-fold model derived by Colvin T. *et al*.^15^ are shown in (**c**,**d**). Color map of residues: red-Q15, grey-L17/L34, blue-M35.

Using solid-state NMR^15, 16^ and other experimental techniques two groups independently resolved recently Aβ_1–42_ fibrils where the individual chains take a S-shaped three-β-stranded form. These fibril models have a two-fold symmetry with the chains packing around residues Q15 and M35. We have shown already in Fig. 2c the structure resolved by Colvin T. *et al*.^15^, called by us the JACS-model. Because of steric constraints the packing in this model cannot be extended to assemblies with higher fold symmetry. However, in previous work^18^ we have proposed, starting with the earlier single fold fibril model of Xiao *et al*.^13^, where the chains have the same S-shaped motif, a fibril assembly with two-fold symmetry that relies on contacts between residue K16 of one chain with residues E22 and D23 of a neighboring chain. This packing, shown in Fig. 3a, can in principle be extended to assemblies with higher fold symmetry, but has not yet been observed in experiments. Hence, in order to test the feasibility of such high-fold assemblies, we first compare through molecular dynamics simulations the stability of our two-fold fibril model with that of the JACS model of Colvin T. *et al*.^15^ (Fig. 3c). As discussed in the method section, we restrict ourselves to fibril models build from Aβ_11–42_ chains since the first ten residues do not take a unique structure in experiments^13^ and were also found be disordered in our earlier molecular dynamic study^18^. For both models, we have generated an initial conformation with six peptides in each fold, corresponding to 12 peptides in each of the two models, as described in the Method section. We refer to these small assemblies as fibrils (and not oligomers) as we could show earlier that six chains per fold is the smallest number that leads to stable fibril-like assemblies^18^. We then follow each of the two systems in two independent molecular dynamics simulations over 200 ns. Start and final configurations are shown in Fig. 3. Using the MM-GBSA approximation we calculate from these runs estimates for the binding energies as listed in Table 1.

**Table 1:** Binding energy (in kcal/mol) distribution for the two-fold model (JACS model) of ref. 15 and the one proposed by us in previous work^18^.

Interaction (kcal/mol)	Our model	JACS-model
vdW	−10.2 (5.7)	−280.3 (11.2)
E-elect	−2018.1 (278.6)	66.6 (16.4)
E-GB	1968.2 (267.8)	44.7 (14.8)
E-surf	−5.9 (0.8)	−32.3 (0.9)
Total	−66.0 (7.1)	−201.4 (12.6)

Each fibril fragment is made of two proto-fibrils with each six layers, see Fig. 3ac.

Both the visual inspection and the binding energies show that for fibrils with two-fold packing symmetry our construction is less stable than the one of the JACS model. As already described by the authors^15^, the inter-chain contact between residue M35(blue) and residues Q15(red) or L17(grey) play a key role for stability of the JACS model. Our calculation also indicates a large contribution of residue L34(grey), and a lesser degree of residues H14/V36/G37/G38 (which all have sidechains not pointing to the packing surface), to the large binding energy in this model. This is because in the JACS model the inter-chain sidechain contacts formed by these residues have better steric packing and larger interaction surface than seen in our model, leading to larger vdW contributions in the JACS model. The resulting lower stability of our model may explain why it has not yet been observed in experiments.

### Comparison of our three-fold Aβ_42_ assembly with an experimentally derived model of Aβ_40_

**Figure 4:**
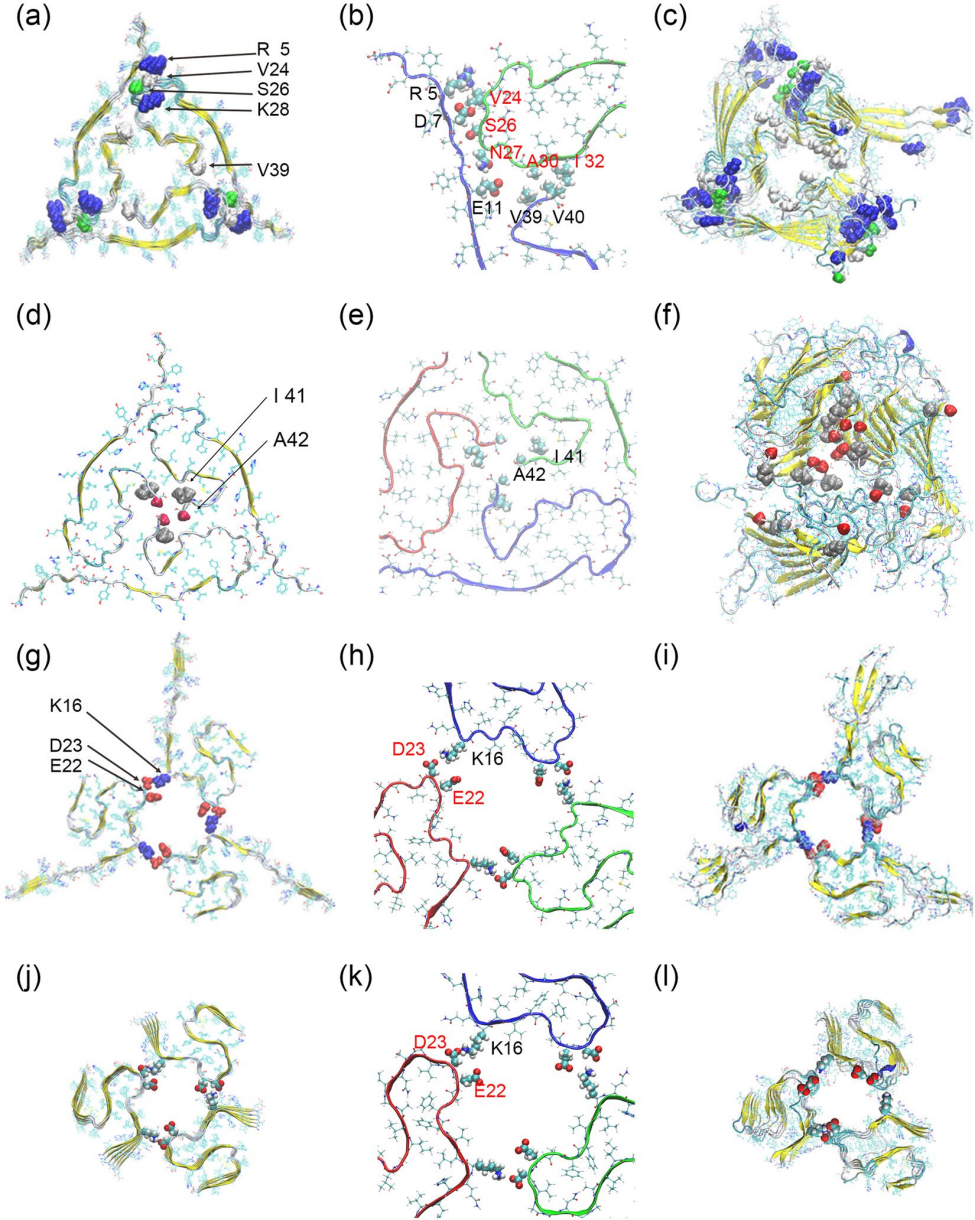
Conformation of three-fold models. Aβ_1–40_ (**a**) initial conformation and (**c**) final conformation after 200 ns of molecular dynamics; the above packing model extended to Aβ_1–42_, (**d**) initial conformation and (**f**) final conformation after 200 ns of molecular dynamics; Aβ_1–42_ three-fold model with our proposed packing, (**g**) initial conformation and (**i**) last conformation after 200 ns of molecular dynamics; the same three-fold model, but for Aβ_11–42_, (**j**) initial conformation and (**l**) last conformation after 200 ns of molecular dynamics. The corresponding side chain packing of the initial conformations are also shown in detail in (**b**,**e**,**h**,**k**).

Only one patient-derived fibril structure has been published so far ref. 11. This structure (PDB-ID: 2M4J), shown in Fig. 4a, has a three-fold symmetry and is made out of Aβ_1–40_ chains which cannot take a S-shaped form but have to assume a U-shaped configuration^25^. The side chain packing of the chains in this assembly is shown in Fig. 4b. It is tempting to assume that the ring-like assembly, differing from other fibril polymorphs, is connected with either toxicity or propagation of amyloids in Alzheimer’s disease^11^. However, no fibrils with three-fold symmetry have been found so far for the supposedly more toxic Aβ_1–42_. An obvious question is whether a three-fold structure such as the patient-derived 2M4J model can also exist for Aβ_1–42_ fibrils.

Taking the patient-derived 2M4J model and adding to all three chains the C-terminal residues I41 and A42 is because of steric constraints only possible if these residues form together with the residue V40 a β-strand, leading again to S-shaped chains. Depending on the direction of the side chains of the two residues I41 and A42 one can construct two structures which differ from the original 2M4J model in that the additional residues fill its pore-like interior. We show in figure Fig. 4d only the more stable one, with the side chain packing displayed in more detail in Fig. 4d. While the other one decayed in less than 20 ns, this one did survive the full 200 ns of our simulation, but became distorted, especially in the pore region (Fig. 4f), and had root-mean-square-deviations to the initial structure that are much larger than the ones seen in our previous simulations of the origin 2M4J model (Fig. 4c). We conclude that the patient-derived three-fold Aβ_1–40_ fibril architecture is incompatible with Aβ_1–42_ peptides because the pores are not large enough for the additional two residues.

Hence, as three-fold fibril structures with U-shaped Aβ_1–42_ peptides are unstable and have not been observed, it is worthwhile to consider arrangements build from S-shaped Aβ_1–42_ chains. Unlike the packing of S-shaped chains observed in the experimentally found two-folded Aβ_1–42_ fibrils^15, 16^, our arrangement can be easily extended to three and higher fold symmetry. Guided by our hypothesis that the higher toxicity of Aβ_1–42_ is due to its ability to take a S-shaped configuration in amyloid assemblies, and assuming that ring-like assemblies (such as the patient-derived Aβ_1–40_ fibril model) are more important than other polymorphs for the pathology of Alzheimer’s disease, we have compared the stability and structural reorganisations of three-fold fibril models of Aβ_1–40_ and Aβ_1–42_. For this purpose, we have run for 200 ns two independent molecular dynamics simulations of a Aβ_1–42_ fibril model with three-fold symmetry generated by us as described by us in the method section and shown in Fig. 4g, with the corresponding side chain packing enlarged in Fig. 4h. Note that such three-fold assembly of S-shaped Aβ_1–42_ peptides cannot be built if one assumes the chain packing seen in the experimentally derived two-fold fibrils (PDB ID: 5KK3). The initial and finial conformations are shown in Fig. 4g,i. There are six layers in both models, and the new simulations follow a similar set-up as in our earlier work^25^ where we studied the stability of the patient derived three-fold Aβ_1–40_ fibril. In order to allow for a meaningful comparison, we also show the initial (Fig. 4a) and finial conformations (Fig. 4c) for this model.

**Figure 5:**
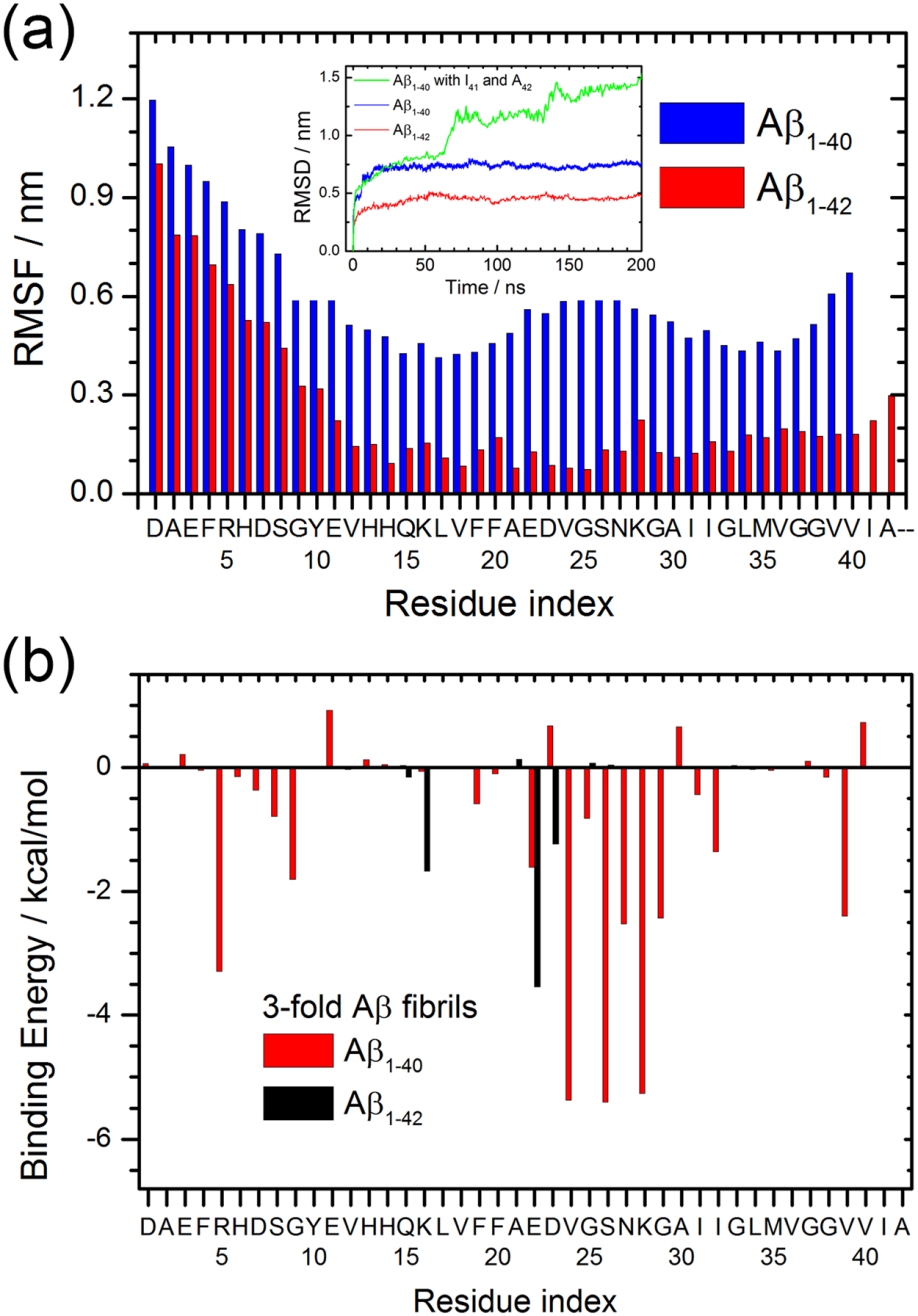
RMSF and binding energy of three-fold model. (**a**) Root-mean-square-fluctuations (RMSF) of residues in the three-fold Aβ_40_ and Aβ_42_ models. The inset shows for both models the root-mean-square-deviation (RMSD) to the start configuration as function of time. (**b**) Comparison of the binding energy of each residue for the two models.

Note that while we arranged for the first ten residues of the Aβ_42_ peptides to form a β-strand in the start conformation, the β-strand secondary structure of these N-terminal residues dissolves over the 200 ns trajectories. However, with this exception the fibril structure remains stable, and the root-mean-square-deviation (RMSD) to the start configuration approaches a plateau after 50 ns as shown in the inset of Fig. 5a. On the other hand, the patient derived Aβ_1–40_ model is much more flexible than our construct, leading to higher RMSD values as seen in the corresponding curve in the inset of Fig. 5a. This is consistent with our previous work^25^, where we also observed a large flexibility in the three-fold patient-derived Aβ_1–40_ model. Various conformational characteristics of both models are listed in Table 2. The three-fold Aβ_1–42_ fibril model has a larger outer diameter (11.4 nm) than the patient-derived Aβ_1–40_ fibril (6.5 nm) which is caused by the arrangement of the N-terminal first ten residues in Aβ_1–42_ model. The inner diameter, equivalent cross-section area and water flow of the Aβ_1–42_ fibril are also larger than that of the patient-derived Aβ_1–40_ fibril, but the difference is smaller than for the outer diameter. We remark that the pore size and water permeability of our Aβ_1–42_ model, exceeding that of the patient-derived Aβ_1–40_ fibril model, could imply a higher toxicity when acting as membrane pores.

**Table 2:** Comparison of three-fold models of Aβ_1–40_ and Aβ_1–42_ fibrils shown in Fig. 4.

Models	Inner diameter (nm)	Outer diameter (nm)	Number of water molecules	Volume (nm^3^)	Equivalent cross-sectional area (nm^2^)	Water flow (nm^−1^)
Aβ_1–42_	2.0	11.4	341.1 (13.8)	10.20 (0.43)	4.10 (0.12)	87.2 (0.2)
Aβ_1–40_	1.7	6.5	263.6 (23.9)	7.88 (0.71)	2.99 (0.19)	77.8 (0.2)

Both three-fold fibril models, the patient-derived Aβ_1–40_ and our proposed Aβ_1–42_ arrangement are stable for at least 200 ns, the length of our simulations. However, the root-mean-square-fluctuation (RMSF) analysis shown in Fig. 5a indicates that our Aβ_1–42_ fibril model is more stable than the patient-derived Aβ_1–40_ fibril, an assumption supported by the lower standard deviation in quantities such as equivalent cross-sectional area that are listed in Table 2. In the patient-derived three-fold Aβ_1–40_ fibril model, the first ten residues adopt a β-strand conformation that appears to be unstable and prone to large fluctuations. In the Aβ_1–42_ models, these N-terminal first ten residues have similar large fluctuations but affect less the overall stability of the fibril.

In order to learn more about the reasons for the differences in stability between the two models, we show in Fig. 5b the binding energy contribution of each residue as approximated by the MM-GBSA approach. In the initial conformation of patient-derived three-fold Aβ_1–40_ fibril model, the packing of the three parts in Fig. 4b is: first, residue R5 with residue D7 and V24 with S26; second, side chain of residue E11 with the backbone of residues G9 and N27; third, residue V39 with residue V40 and A30 with I32. For most parts, these contacts also add later to the binding energy. The binding energy between the fold components is more favorable in the patient-derived Aβ_1–40_ model than in the Aβ_1–42_ model, indicating that the stability differences do not result from contributions at the packing surface but from the intra-chain packing pattern. The S-shape architecture of Aβ_1–42_ chains is more stable than the U-shape form of the Aβ_1–40_ peptides.

### Stability of our proposed N-fold Aβ_42_ models

Scanning tunneling microscopy (STM) and atomic force microscopy (AFM) measurements allow one to estimate the diameter of fibrils. For Aβ_40_ and Aβ_42_ fibrils this diameter varies over a broad range from 3 nm to more than 20 nm. Single fold and two-fold models cannot describe fibrils with a diameter of larger than 10 nm, hence, the experimental data suggest that some of the fibrils are built from more than two protofibrils, i.e., have three and higher fold symmetry. An example is the patient-derived fibril model 2M4J made from U-shaped Aβ_1–40_ chains. In principle, its packing can be extended to higher fold numbers, however, this would even further reduce its stability. Unlike U-shaped Aβ_1–42_ peptides, S-shaped Aβ_1–42_ chains can in principle also assemble into fibrils with fold symmetry larger than three when packing as proposed by us. However, it is not clear whether such assemblies are stable or have desirable structural properties. In order to investigate these questions, we have generated a series of Aβ_11–42_ fibrils with three, four, five, six and 12-fold symmetry, and have followed their evolution in two independent molecular dynamics simulations of 200 ns (50 ns for the 12-fold fibril). In order to explore the role of the first ten residues, which are disordered in the experimental structures, on the stability of the N-fold symmetric assemblies, we have also repeated these simulations for full-sized Aβ_1–42_ with either three-fold or six-fold symmetry, using the same number of chains in each fibril fragment and the same simulation protocol. Analyzing these control simulations, we find that the first ten residues become disordered even if they are arranged initially as a β-strand, but the overall stability and evolution of the fibrils differ little from the corresponding Aβ_11–42_ fibrils. This can be seen by comparing Fig. 4g and i (start and final configuration for full-sized chains) with Fig. 4j and l (start and final configuration for the truncated peptides). Note that the side chain packing is the same for both models (Fig. 4h and j). Note also, that quantities such as pore size or RMSD to the start configuration are the same within the statistical errors. Hence, in the following we only consider Aβ_11–42_ fibrils as the smaller size reduces the computational costs.

**Figure 6:**
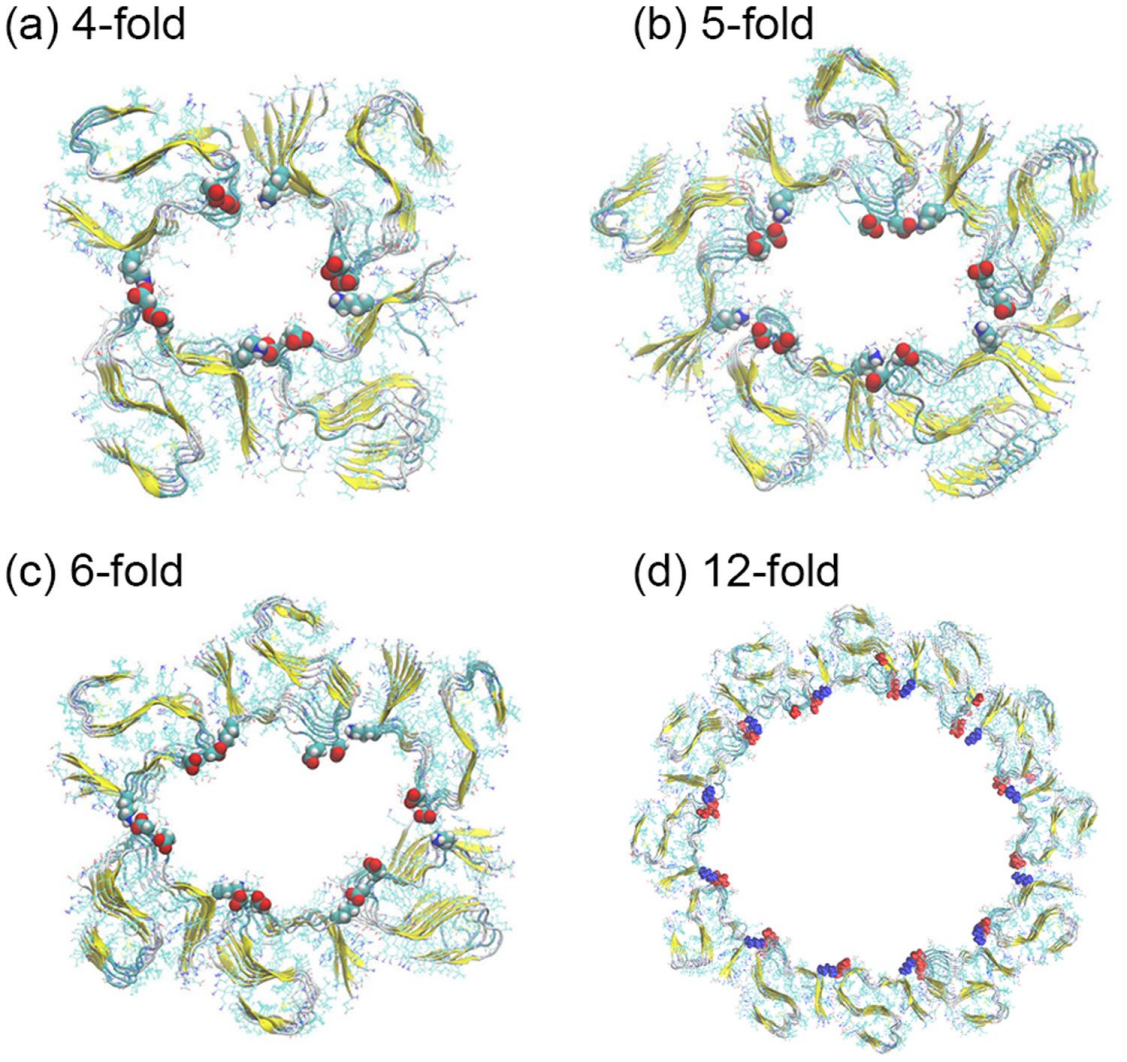
Final conformations of Aβ_11–42_ N-fold fibril models after molecular dynamics simulations of 200 ns (50 ns in the case of the 12-fold model). The side chains of K16, E22 and D23 are shown as vdW spheres.

All of our N-fold models are stable over the 200 ns of the trajectories (50 ns for the 12-fold fibril). From the two independent runs, we have chosen the final configuration with the largest RMSD to the start configuration, and show it for the four, five, six and 12-fold case in Fig. 6. The corresponding three-fold structure is already shown in Fig. 4. In all cases, the N-fold models maintain their backbone H-bonds and contacts between the packing surfaces.

**Figure 7:**
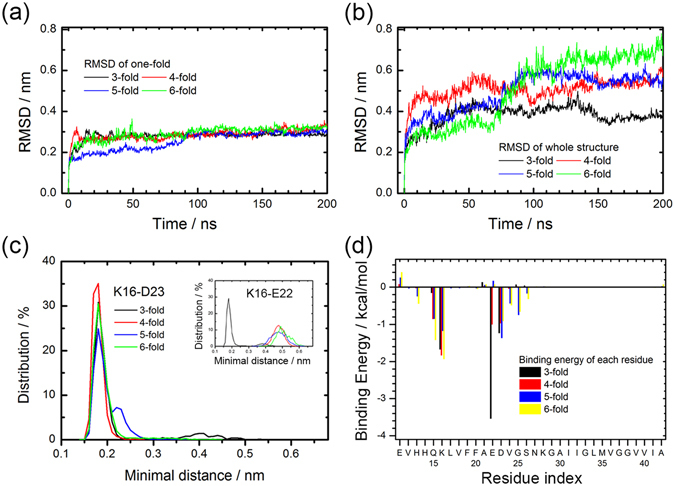
Conformation characteristics and binding energy of N-fold model. RMSD with respect to the start configuration as function of time for a single chain (**a**) and the whole model (**b**). The minimal distance between corresponding inter-chain residues K16 and D23, with the insert showing the distance between residues K16 and E22 **(c)**. Binding energy decomposition of each residue between different chains in N-fold models (**d**).

We have calculated for each fold the RMSD of a *single chain* to the corresponding chain in the start configuration, and have plotted that quantity as function of time in Fig. 7a. The corresponding RMSD values for the whole model are shown in Fig. 7b. Similar to the previously shown two-fold and three-fold models, both RMSD values approaches a plateau after 20 ns indicating that equilibrium has been reached. The RMSD evolution of the single chains in Fig. 7a resembles closely the one observed in earlier work for single-fold fibrils^18^. Comparing the RMSD of the whole models (Fig. 7b) and that of single chains (Fig. 7a), we see that the large RMSD observed for all of the N-fold models are caused by the relative movement between chains while the individual chains change little and keep their S-shape motif. For instance, in the six-fold model, each chain maintains stable during simulations, and the fluctuation seen in Fig. 7b is caused by the various chains changing their relative position. This can be also seen in Fig. 7c where we plot the distribution of the distance between the side-chains of residue K16 and residue E22 or D23. The peak in the two distributions indicates that the inter-chain salt-bridge persists during the simulation, but as the fold number N increases, the salt-bridge between residues K16-E22 (Fig. 7c) is gradually lost and the distance between the two residues shifts to a value of 5 Å, while it stays at 2 Å, a value more typical for a salt bridge, for the distance between residues K16-D23 (Fig. 7c).

The difference in the distance distributions is related to the different binding energies per residue shown in Fig. 7d. Besides the inter-chain K16-E22/D23 salt-bridge, the residues Q15, V24 and G25 are also in contact with other chains. In the two and three-fold models, the electrostatic interactions between residues E22 and K16 are stronger than the ones between residues K16 and D23. In the higher fold models, the packing angles between the side chains of residue K16 and either residue E22 or D23 changes and the two sets of interactions have similar strength. As these salt bridges are the essential for our proposed models, we would expect that the packing depends on pH or salt-concentration. On the other hand, these salt bridge could be easily detected by NMR and similar methods, even without resolving the structures in detail.

As discussed above, experimentally measured fibril diameters suggest the existence of fibrils with three and higher-fold symmetry. These measurements can be compared with the outer diameter of our models, allowing us to verify that our models are reasonable (i.e., are compatible with the experiments). For this purpose, we define the outer diameters of the N-fold models as the maximal distance between any pair of carbon-alpha atoms in the residues E11 of chains located in the same layer, and we show the corresponding values for the various N-fold models in Table 3. Note that for the six-fold models of Aβ_11–42_ we find a distance of about 10 nm which well agrees with experimental measurements of Aβ channel on membrane (13.2 nm for six-fold channel)^19^ or 12–24 mer of Aβ oligomers (11.2–14.3 nm in height)^26, 27^. The radial symmetry of the proposed fold models leads also to formation of an inner pore inside the fibrils whose size can be approximate by measuring in our models the maximal distance between any pair of carbon-alpha atoms in residues K16 of all chains in the same layer. These estimates are also shown in Table 3.

**Table 3:** Inner and outer diameters of our N-fold fibril models as defined in the text.

Models	Inner diameter (nm)	Outer diameter (nm)
3-fold	1.9	6.3
4-fold	2.5	8.3
5-fold	3.9	9.1
6-fold	5.2	10.6
12-fold	11.8	17.3
Aβ_1–42_: 3-fold	2.0	11.4
Aβ_1–42_: 6-fold	5.1	15.2

**Figure 8:**
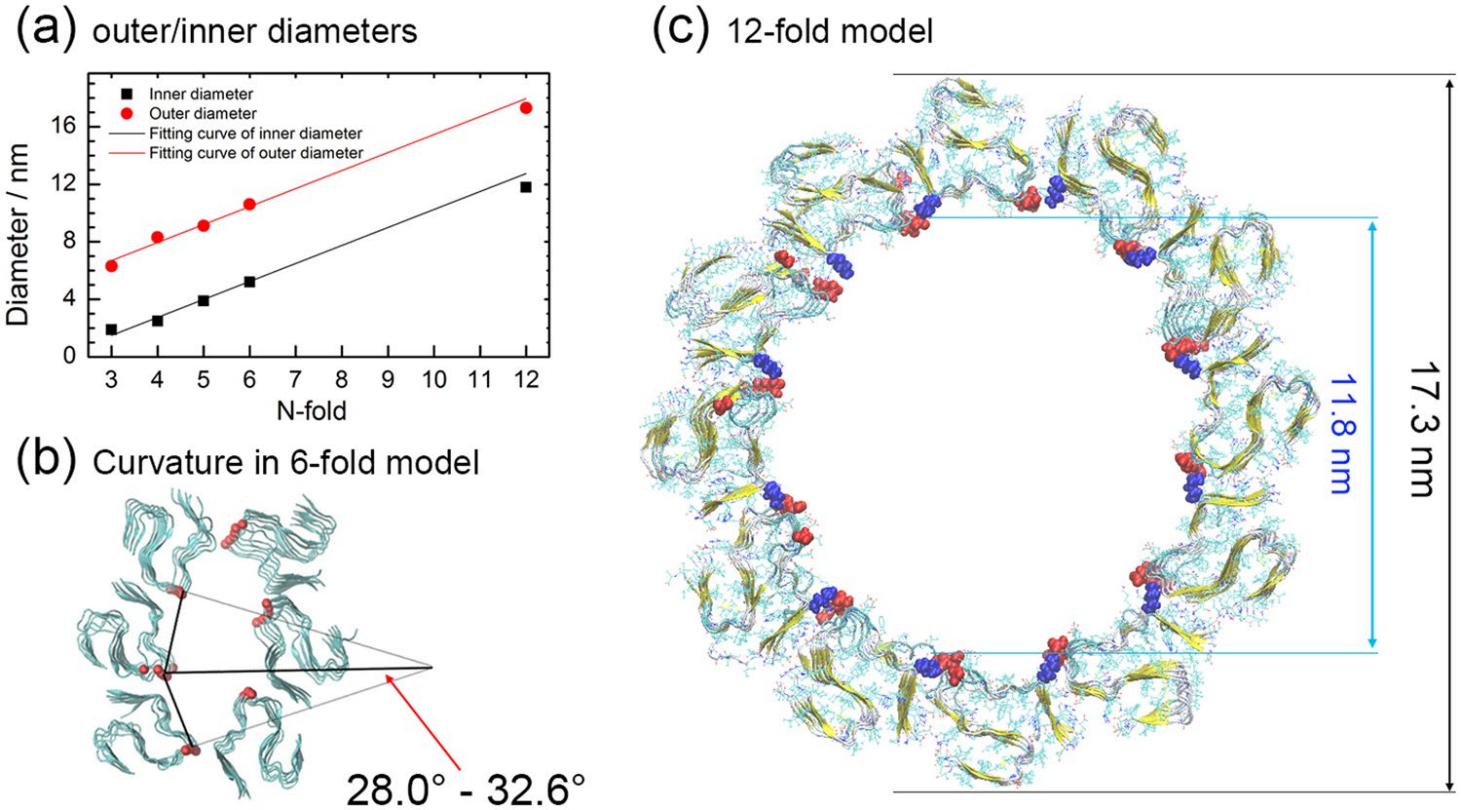
Outer and inner diameters. (**a**) The outer and inner diameters of N-fold models and the corresponding fit as function of fold number. (**b**) Curvature of the adjacent fold component in the six-fold model. (**c**) The final confirmation of the 12-fold model after 50 ns of molecular dynamics simulation, and its inner and its outer diameters.

The pore size increases with fold number and is about 5.2 nm for six-fold models built from the full-length peptide Aβ_1–42_. Comparing with that of common water channels, for example, AQP1 which have effective pore diameter around 0.3–0.4 nm^28^, the pore inside the six-fold fibril are much more favorable of permeation. In a similar way, the outer diameter increases with fold number to about 15.2 nm for 6-fold models of Aβ_1–42_, fitting well the experimental measurements. In both cases, the growth can be fitted by a straight line (see Fig. 8a) by

1 $${D}_{outer}(N)=1.25\times N+2.95$$

2 $${D}_{inner}(N)=1.25\times N-2.25$$

Here D_outer_ (D_inner_) is the outer (inner) diameters of fibrils, and N is the fold number of the models. We expect that the slope of the scaling relations for the two diameter is the same for sufficiently large N. For this reason, we have fit our data with the same slope. Note, that we did not use our 12-fold data for the fit, allowing us to use these points to check the predictive power of our fit. According to our fitting equation, for the N = 12 fold, the outer diameter will be 18 nm, and the inner diameter will be 12.8 nm. In order to check the prediction, we constructed the 12-fold models of Aβ_11–42_ and perform a 50 ns molecular dynamics run. The final configuration is shown in Fig. 8c. The fibril kept stable in such time scale and we find values of 11.5 nm for the inner diameter and 17.4 nm for the outer diameter. As also seen in the scaling plot in Fig. 8a, the 12-fold data are compatible with the fit, but the simple linear fit seems to overestimate the diameters. This is because the cross-sectional area of the N-fold models is better described by an ellipse than a circle, and leading to deviations from the linear function on higher fold numbers. This break down of the fitting relation may actually indicate that there is an optimal size of our N-fold assemblies. In order to demonstrate this point, we show in Fig. 8b for the six-fold model the dominant inter-layer interactions (linked by black line). Their curvature can be described by an angle of about 28–34 degrees, which suggests that the best packing N-fold models would be 11–13-mers and would have a radius of curvature of about 5–6 nm. This assumption seems to be supported by our results from the 12-fold simulation. For instance, the β1 section and turn1 section on adjacent peptides form contacts that are in all twelve components better than seen in the five-fold and six-fold models. Interestingly, unlike the six-fold model which has an elliptical pore, the 12-fold model has a nearly circular pore which varies less than 1 nm along the 50 ns trajectory. However, these simulations may be too short to examine rigorously the stability of the 12-fold model. We also remark that for the full length Aβ_1–42_ chains, the outer diameters would be 5 nm larger than for the Aβ_11–42_ peptides, leading to an outer diameter of about 23–24 nm for 12-fold Aβ_1–42_ fibrils. i.e., diameters that are at the upper range of the experimental measurements.

**Figure 9:**
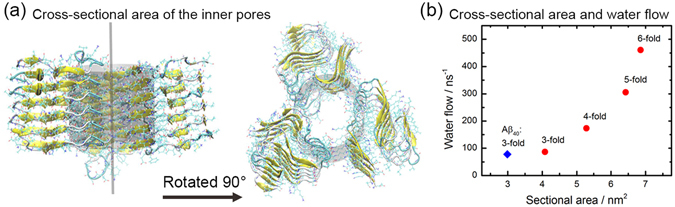
Pores and water flow. (**a**) Cross-sectional area of the inner pores in our N-fold models. (**b**) The cross-sectional area of pores and water flow in our N-fold fibril models.

All of our models are built out of six layers. The height of fibrils along the z-axis (shown in Fig. 9a) are similar in all models. For example, the average height of fibrils in three-fold models is about 2.5 nm, where we define the height as the average distance between corresponding carbon-alpha atoms of residues in the first and sixth layer. The average distance between two peptides in our models is about 5 Å, which is a common value for the distance between the main chains of two peptides in a parallel β-sheet. Since the cross-sectional area is not regular, it is hard to calculated it directly. Instead, we have estimated the volume of the pores from the number of water molecules number inside it (shown in Table 4), assuming that the average occupied volume of each water molecule is about 2.99 × 10^−2^ nm^3^ at room temperature, and divided the so-calculated volume by the height of the fragment to derive the equivalent cross-sectional areas that are shown in Table 4.

**Table 4:** Average number of water molecules in pores and the equivalent cross-sectional area as measured in our Aβ_11–42_ fibril models.

Models	Number of water molecules	Volume (nm^3^)	Equivalent cross-sectional area (nm^2^)	Water flow (nm^−1^)
Three-fold	338.5 (15.7)	10.12 (0.47)	4.08 (0.12)	85.4 (1.2)
Four-fold	438.39 (13.20)	13.11 (0.39)	5.28 (0.10)	172.4 (0.8)
Five-fold	533.0 (15.9)	15.9 (0.5)	6.4 (0.1)	297.3 (8.2)
Six-fold	568.6 (10.9)	17.0 (0.3)	6.9 (0.1)	454.4 (6.4)
Aβ_40_ Three-fold	263.6 (23.9)	7.9 (0.7)	3.0 (0.2)	77.8 (0.2)

Note that the diameters of the pores in our models are larger than some of the ion channel, for example, that of potassium channels which are about 1.2 nm^29, 30^, suggesting water leakage through such fibrils inserted in cell membranes as a possible toxicity mechanism. If such N-fold fibrils insert themselves into the membrane, they would form pores that disrupt the osmotic pressure of membranes. The thickness of a typical bilayer lipid is about 3–4 nm which means an N-fold fibril with 6–8 layers could cross it as the distance between opposite residues in β-strand is about 5 Å. In order to estimate the effectiveness of such pores we have also calculated the water flow in our models, where the pores are defined as described above, and only water molecules crossing through the fibril either upwards or downwards are counted. The average number of water molecules permeating through the pores per nanosecond is shown for each model in Table 4. The relationship between cross-section area and water flow are plotted in Fig. 9b. As expected, we find that the larger of the pores, the better is permeation. And the water flow is a quadratic function of pore size.

Previous AFM experiments have probe the structures of Aβ_42_ channels in membranes^19^. Interestingly, not a single kind pores was found, but channels with three, four, five and six subunits are observed. The measured diameter for channels made of four subunits is 11.5 nm and 18.1 nm for channels made of five subunits. These values are compatible with the outer diameters of the corresponding N-fold models developed by us. Hence, our ring-like assemblies may serve as models for the experimentally observe neurotoxic Aβ_42_ channels in membranes^19, 31^. Note, however, that the structure of these assemblies is difficult to resolve in experiments, and other models, such as beta-barrels have been proposed for the N-subunits Aβ_42_ channels^19, 32^, with no evidence in favor of either model. We also remark that our three-fold model with four layers is compatible in height and size with the neurotoxic oligomers of Aβ_42_ called large fatty acid derived oligomers(LFAOs)^33, 34^ that consist of 12 or 24 peptides and have a height of about 5–7 nm.

## Discussion

The research presented in this article is guided by two hypothesizes. First, we conjecture that the higher toxicity of Aβ_42_ when compared to that of Aβ_40_ is due to the ability of Aβ_42_ peptides to assemble not only into fibrils made of U-shaped chains but in addition also in such build out of three-stranded S-shaped chains. Our second assumption is that toxicity of amyloids is related to them forming pore-like structures. In the present paper, we demonstrate that S-shaped Aβ_42_ peptides can indeed assemble into ring-like structures that are stable over the length of our molecular dynamic simulations.

Our proposed models rely on a chain packing that is stabilized by inter-chain salt bridges between residues K16 and either E22 or D23. This packing is energetically less favorable than the one stabilized by contacts between residues Q15 and M35 that has been observed in the experimentally resolved Aβ_42_ fibrils with two-fold symmetry^15, 16^. In passing, we remark that the two binding forms could be compatible. One could imagine a protofibril to be formed by Q15-M35 contacts, and assemble with two other such protofibrils through inter chain salt bridges formed between residues E22 and D23 or K16 that would lead two fibrils growing not only along the fibril axis but also perpendicular to it which may open applications for the design of new materials. We have not pursued this idea and instead focused on the main advantage of our proposed packing through inter-chain salt bridges between residues K16 and either E22 or D23, namely that it is not sterically constraint two two-fold assemblies. Three and higher fold assemblies with our chain packing lead naturally to pore-like structures. Geometrical considerations suggest 12-fold models as the optimal arrangement, however, already our three-fold Aβ_1–42_ fibril model is more stable than the patient-derived Aβ_1–40_ fibril model (PDB-Id: 2M4J) that has the same three-fold symmetry. Similarly, N-fold assemblies with N = 4, 5, 6 and 12 are also stable and have outer diameters that are comparable with some of previous experimental measurements of fibril diameters. As our assemblies are characterized by a unique pattern of inter-chain sidechain electrostatic interactions between residues K16 and either E22 or D23, they could be experimentally identified by the resulting specific NMR signal. We also suspect that fibrils with our arrangement would have a strong response to changes salt concentration or pH.

All of our ring-like N-fold fibril models have an interior pore through which water can flow, suggesting as mechanism of cell toxicity membrane leaking when the fibrils are inserted into bilayer lipids. In size and biophysical characteristics they are consistent with neurotoxic Aβ_42_ channels observed with AFM experiments in membranes^19, 31, 35^. We conjecture that our three-fold model with four layers could serve as a model for the 12–24 residue large fatty acid derived oligomers(LFAOs)^33, 34^ whose neurotoxicity has been recently shown in both cell culture^33^ and in a mice brains^36^. We now plan to test this hypothesis experimentally in a collaboration with the Ranagchari Lab that we hope to start soon. We emphasize that our N-fold models are different from previous β-barrel models of Aβ peptides^19, 32^ and point out another possible mechanism for toxicity of Aβ_42_ fibrils or oligomers. As Aβ_40_ peptides cannot assume the triple β-strand motif, they cannot form the pore-like N-fold fibril structures that we have proposed in this study. This difference therefore could explain the higher toxicity of Aβ_42_ amyloids over that of Aβ_40_.

Our investigation touches a general problem in amyloid studies, namely the role of polymorphism. In order to minimize the exposed hydrophobic surface, the Aβ-chains have to collapse into U-shaped or S-shaped packing conformations leading already to a plethora of polymorphic structures^37^. However, through domain-swapping the peptides can also adopt extended conformations and hide the hydrophobic regions into the packing surface^17^. The domain-swapping region located on C-terminal region of Aβ_42_ and it formed three extended β-sheet-like section (15–22, 28–36 and 39–42)^17^ which is similar with the Aβ_42_ S-shape model^13^. The domain-swapping model would have great hydrophobic exposed area in single-fold condition, which is differs from the JACS model that the fibril could also be existed in single-fold S-shape conformation. The variations in architectures of two-fold models suggest that high-fold model of fibrils are not just simple rotation and combination of stable single-fold fibril models, for example, U-shape or S-shape in Aβ_42_ fibrils. We cannot exclude the possibility that the resolved fibril models are just particular cases, and that there are multifarious mechanisms of forming high-fold fibrils instead of a unique one.

## Methods

### Model construction

In our previous work^18^, we have proposed that the newly found S-shaped Aβ_1–42_ peptides can form two-fold fibrils by packing two chains in such a way that an inter chain salt bridge between residue K16 and residues E22 or D23 is formed. This packing pattern, called PSA by us, can be extent to fibrils with higher-fold rotational symmetry. For the present study, we build three, four, five, six and 12-fold models of S-shaped Aβ_11–42_ fibril models with six peptides in each layer. While these assemblies are technically not fibrils but oligomers, we use the term “fibril” throughout this paper as we found earlier that six chains is the minimal number that leads to stable single-fold fibril-like assemblies^18^. Focusing on residues 11 to 42 is justified because the first ten residues do not take a unique structure in experiments^13^ and were also found be disordered in our earlier molecular dynamic study^18^. However, in order to exclude any bias resulting from omitting the first ten residues, we have also constructed high-fold models built out of full-length Aβ_1–42_ peptides, specifically, three-fold and six-fold models where the first ten residues adopt a β-strand conformation. As our molecular dynamic simulations of these two systems (see Result section) showed that the first ten become quickly disordered, we used for all other simulations the shorter and computationally less costly Aβ_11–42_ fibril models.

All our N-fold fibrils are built using AmberTools^38^ as follows: first, we selected from our previous molecular dynamics work^18^ a representative conformation of a single-fold S-shape Aβ_11–42_ fibril fragment. Second, this six-layer fragment was replicated and, through rotation and translations, the protofibril fragments arranged in a way that residue K16 is close to residues E22 or D23 of another chain. The resulting n-fold structures were minimized in a third step and allowed to relax in 10 ns molecular dynamics simulation using Amber14^38^, restraining the inter- chain contacts between residue K16 and residues E22 and D23. The so-obtained structures were chosen as start configurations for our molecular dynamics simulations. The procedure was similar for the Aβ_1–42_ fibril models, only that in the first step also the first ten residues were added to each chain, assuming a perfect β-strand configuration.

Using more recent experimental results, two other groups also developed two-fold models of S-shape Aβ_42_^15, 16^. For this reason, we compare our two-fold model also with the two-fold Aβ_11–42_ fibril model (PDB ID: 5KK3) developed by Colvin T. *et al*. which we name JACS-model in our present work. For this purpose, the PDB structure was truncated to a six-layer system comparable to our other models and studied in our simulations with the same parameters and setup.

### Molecular dynamics simulation

Our simulations rely on the GROMACS 4.6.7 tool suit^39^, using the CHARMM 36 force field^40^ and TIP3P solvent^41^. For each model, two independent trajectories are followed for 200 ns, starting from the same initial conformation but different, randomly chosen initial velocities corresponding to a temperature of 310 K. This temperature and a pressure of 1 bar are controlled by v-rescale thermostat^42^ and Parrinello-Rahman barostat^43^. The LINCE algorithm^44^, SETTLE algorithm^45^ are employed for bonds so that the time step of 2 fs are used for integration. The particle mesh Ewald(PME) method^46^ are used to calculated electrostatic interactions. The cutoff of van der Waals(vdW) and electrostatic interactions are 1.4/1.0 nm. For 3/4/5/6-fold of Aβ_11–42_ fibrils, the cubic boxes are used and the corresponding box size are 9.86/10.70/11.43/12.74 nm. The boxes are large enough so that the peptides do not have direct interactions with their images.

Since the 12-fold model contains 2304 residues, we tried to minimize the computational costs by choosing a triclinic box with periodic boundary conditions, constraining the fibril rang into x-y plane with a weak position restraint of 12.0 kJ/nm on z-axis for carbon alpha of all valine residues. This restraint ensured that the fibril did not have direct contact or electrostatic interaction with its image and greatly reduced the box size and number of water molecules, but we still had to restrict our simulations of this system to 50 ns.

In order to exclude force field related biases, and to connect with our earlier work^18^, we have also simulated the three-fold model with the same set-up, but using the Amber ff99SB-ildn force field^47^. As the simulations of this system differed little from the one using CHARMM36, we used only the later in all other of our simulations.

For most of our analysis we discard the first 50 ns of our simulations and the presented data are averaged over the remaining 150 ns and two trajectories. The binding energies are approximated through MM-GBSA using the MMGBSA.py tools^48^ in Amber 14, where we employ the generalized Born (GB) model developed by H. Nguyen *et al*.^49^ and do not consider entropic contributions. The water flow analysis is carried out with an in-house plugin to VMD that has been developed in our group and is available from the authors.

### Data Availability

The datasets generated during and/or analysed during the current study are available from the corresponding author on reasonable request.
